# Perspectives on Home Time and Its Association With Quality of Life After Inpatient Surgery Among US Veterans

**DOI:** 10.1001/jamanetworkopen.2021.40196

**Published:** 2022-01-11

**Authors:** Shipra Arya, Ashley H. Langston, Rui Chen, Marzena Sasnal, Elizabeth L. George, Aditi Kashikar, Nicolas B. Barreto, Amber W. Trickey, Arden M. Morris

**Affiliations:** 1 Division of Vascular Surgery, Stanford University School of Medicine, Stanford, California; 2 Stanford-Surgery Policy Improvement, Research, and Education Center, Palo Alto, California; 3 Surgery Service Line, Veterans Affairs Palo Alto Healthcare System, Palo Alto, California

## Abstract

**Importance:**

Home time, defined as time spent at home after hospital discharge, is emerging as a novel, patient-oriented outcome in stroke recovery and end-of-life care. Longer home time is associated with lower mortality and higher patient satisfaction. However, a knowledge gap exists in the measurement and understanding of home time in the population undergoing surgery.

**Objectives:**

To examine the association between postoperative home time and quality of life (QoL), functional status, and decisional regret and to identify themes regarding the meaning of time spent at home after surgery.

**Design, Setting, and Participants:**

This mixed-methods study including a survey and qualitative interviews used an explanatory sequential design involving 152 quantitative surveys followed by in-depth interviews with 12 participants from February 26, 2020, to December 17, 2020. US veterans older than 65 years who underwent inpatient surgery at a single-center veterans hospital within the prior 6 to 12 months were studied.

**Exposures:**

Quality of life, measured by the Veterans RAND 12-item Health Survey and 19-item Control, Autonomy, Self-realization, and Pleasure scale; functional status, measured by activities of daily living (ADL) and instrumental ADL scales; and regret, measured by the Decision Regret Scale.

**Main Outcomes and Measures:**

Home time, standardized as percentage of total time spent at home from the time of surgery to the time of survey administration. Associations between home time and QoL, function, and decisional regret in the survey data were analyzed using Spearman correlation in the overall cohort and in operative stress score subcohorts (1-2 [low] vs 3-5 [high]) in a stratified analysis. The 12 semistructured interviews were analyzed to elicit patients’ perspectives on home time in postoperative recovery. Qualitative data were coded and analyzed using content and thematic analysis and integrated with quantitative data in joint displays.

**Results:**

A total of 152 patients (mean [SD] age, 72.3 [4.4] years; 146 [96.0%] male) were surveyed, and 12 patients (mean [SD] age, 72.3 [4.8] years; 11 [91.7%] male) were interviewed. The median time to survey completion was 307 days (IQR, 265-344 days). The median home time was 97.8% (IQR, 94.6%-98.6%; range, 22.2%-99.5%). Increased home time was associated with better physical health–related QoL in the Veterans RAND 12-item Health Survey (*r* = 0.33; 95% CI, 0.18-0.47; *P* < .001) and higher ADL scores (*r* = 0.21; 95% CI, 0.06-0.36; *P* = .008) and instrumental ADL functional scores (*r* = 0.21; 95% CI, 0.04-0.37; *P* = .009). Decisional regret was inversely associated with home time in only the high operative stress score subcohort (*r* = −0.22; 95% CI, −0.47 to −0.04; *P* = .047). Home was perceived as a safe and familiar environment that accelerated recovery through nurturing support of loved ones.

**Conclusions and Relevance:**

In this mixed-methods study including a survey and qualitative interviews, increased home time in the first year after major surgery was associated with improved daily function and physical QoL among US veterans. Interviewees considered the transition to home to be an indicator of recovery, suggesting that home time may be a promising, patient-oriented quality outcome measure for surgical recovery that warrants further study.

## Key Points

**Question:**

What are patients’ perspectives about home time after surgery, and is home time associated with quality of life, function, and decisional regret after surgery?

**Findings:**

In this mixed-methods study including a survey of 152 US veterans and 12 qualitative interviews, increased home time in the first year after major surgery was associated with improved daily function and physical quality of life. Patients considered home as the preferred place for postoperative recovery, and qualitative interviews suggested the importance of home time in mental and emotional recovery from surgery.

**Meaning:**

The findings suggest that home time is a potentially useful patient-oriented surgical quality outcome measure.

## Teaser

This mixed-methods study including a survey and qualitative interviews of US veterans who underwent inpatient surgery assesses whether the percentage of time spent at home after surgery was associated with quality of life, function, and decisional regret after surgery.

## Introduction

Home time is emerging as a novel patient-centered outcome defined as the time a patient spends outside a hospital, skilled care facility, or nursing facility after a health care event.^1^ Specifically, home time has proved to be a useful outcome measure in stroke recovery,^2,3^ heart failure,^4,5^ and end-of-life care.^1^ Among stroke survivors, home time was associated with modified Rankin Scale scores at 90 days and 1 year^2^ and varied by hospital annual stroke volume, severity of case mix, and region.^3^ Home time also has been shown to be a surrogate measure for quality of life (QoL), disability, and functional recovery after stroke.^6,7,8^ Among patients with heart failure, 30-day home time has been shown to be a quality metric associated with lower readmissions and mortality as well as reclassified hospital performance compared with other risk-adjusted measures.^5^ Days at home may be especially important for patients receiving end-of-life care because they value time spent in familiar environments instead of hospitals or skilled care services,^1^ and it is plausible that this feeling extends to the postoperative period for patients who undergo high-risk surgery.

A previous study^9^ found that non–home discharge after surgery is associated with higher mortality, especially among patients with frailty. An analysis^10^ of Medicare beneficiaries undergoing cancer surgery also showed a substantial amount of time spent away from home in the hospital and/or nursing home. However, there is a lack of measurement and understanding of home time in the population undergoing surgery and its association with postoperative recovery and QoL.

To explore the importance of home time in the postoperative period, we conducted a mixed-methods study including a survey and qualitative interviews using an explanatory sequential design^11^ of US veterans after they had undergone inpatient surgery. We examined the association between postoperative home time and functional status, QoL, and decisional regret and conducted semistructured interviews of purposively sampled patients. We hypothesized that longer home time would be associated with better QoL and functional recovery after surgery and would be inversely associated with decisional regret. We sought to identify themes regarding the meaning of time spent at home after surgery in qualitative interviews to understand how patients valued recovering at home.

## Methods

### Study Sample

This prospective, mixed-methods study including a survey and qualitative interviews using an explanatory sequential design^11^ (eFigure in the Supplement) was conducted from February 26, 2020, to December 17, 2020, and included US veteran patients older than 65 years who underwent an elective inpatient surgical procedure at the Veterans Affairs Palo Alto Health Care System within the previous 6 months to 1 year. These criteria yielded 315 patients in the eligible cohort (50.8% recruitment response rate and 74.2% contact rate), in accordance with the American Association for Public Opinion Research (AAPOR) reporting guideline.^12^ Surveys were administered in person (clinical intercepts) or by telephone to assess (1) quality of life (QoL), (2) functional status, and (3) decisional regret about undergoing surgery. We first analyzed the survey results and, based on these data, purposively sampled 6 patients with high home time (HHT; 95.7%-99.3%) and 6 patients with low home time (LHT; 43.3%-90.1%) for in-depth semistructured interviews. The interview guide was developed to explain and provide context for the initial quantitative results.^13^ The sequential study design thereby generated a more comprehensive understanding of studied phenomena and enhanced the validity and credibility of the study.^11^ Verbal informed consent was obtained from the 152 survey participants and the 12 interview participants as outlined in the study protocol approved by the Stanford institutional review board and Veterans Affairs Palo Alto Health Care System Research and Development Committee. All data were deidentified. This study followed the Standards for Reporting Qualitative Research (SRQR) reporting guideline.

### Measurement Strategy

#### Dependent Variable

Home time was calculated as the percentage of time from the surgical procedure date to the survey date that participants spent at home. For example, if a patient had an inpatient stay for 7 days after surgery and 21 days at a skilled nursing facility followed by no other hospitalizations and the total follow-up period to the survey date was 200 days, the home time percentage was calculated as 170/200 = 85%. Inpatient days and non–home stay days (eg, rehabilitation facility or hospitalization) were obtained from the Corporate Data Warehouse inpatient administrative data tables and were cross-validated using medical record review.

#### Surveys

Surveys were used to capture QoL, functional status, and decisional regret (about undergoing surgery) at a single time point between 6 months and 1 year after surgery. Quality of life was assessed using (1) the Veterans RAND 12-item Health Survey (VR-12)^14^ with the physical component score (PCS) and mental component score (MCS) subscales and (2) the 19-item Control, Autonomy, Self-realization, and Pleasure scale (CASP-19)^15^ survey with 4 subscale domains that make up the survey’s name. Functional status was measured using the Katz Index of Independence in Activities of Daily Living (ADL)^16^ and the Lawton/Brody Instrumental Activities of Daily Living (IADL)^17^ scales. We assessed regret about the surgery with the Decision Regret Scale (DRS).^18^

#### Covariates and Comorbidities

Patient age, sex, race and ethnicity, surgical service, admission to a skilled nursing facility, and comorbidities (presence or absence of diabetes, hypertension, hyperlipidemia, congestive heart failure, chronic kidney disease, and chronic obstructive pulmonary disease) were assessed using Corporate Data Warehouse and medical record review. Patients were considered to have the condition if any of the relevant *International Classification of Diseases, Ninth Revision, Clinical Modification* or *International Statistical Classification of Diseases, Tenth Revision, Clinical Modification* codes (eTable in the Supplement) were listed in any position for any outpatient or inpatient visits within 1 year before their surgical procedure.^19^

#### Stratification Variable

To account for case complexity and differences in expected postoperative recovery, we stratified the cohort based on operative stress score (OSS). Two subcohorts were created by grouping lower stress procedures (OSSs of 1-2 [low OSS]) and higher stress procedures (OSSs of 3-5 [high OSS]).

### Quantitative Analysis

The correlation between home time and QoL, functional status, and decisional regret was analyzed using Spearman rank correlation in the overall cohort and then in stratified samples of low OSS and high OSS subcohorts. Patients with missing ADL, IADL, CASP, and DRS values were excluded from the correlation tests. Statistical significance was assessed at a 2-sided α = .05. All quantitative analyses were conducted using SAS software, version 9.4 (SAS Institute Inc).

### Semistructured Qualitative Interviews

#### Data Collection

Semistructured interviews were conducted by telephone or videoconference with a subset of the survey participants (n = 12) using a purposeful sampling strategy (6 had a high percentage of home time and 6 had a low percentage of home time after their surgical procedures). Interviews were conducted by 2 of us (A.H.L. and M.S.).

The interview questions aimed to explain and confirm or clarify the key findings from the surveys by capturing patients’ perspectives about the meaning and value of home time in postoperative recovery. The interview guide included open-ended questions to elicit respondents’ mental and physical health and activities, the desired role and autonomy in the recovery process, the meaning of home, and the value of being at home during recovery.

The interviews were conducted by both interviewers: one led the conversation while the other took thematic notes and asked clarifying questions. Immediately after each interview, the interviewers met to discuss first impressions and major themes, and then each interviewer separately wrote memos summarizing their observations.

#### Qualitative Coding and Analysis

We used a modified thematic analysis approach^20^ to analyze the qualitative data. Interviews were audiorecorded, transcribed verbatim, and coded by 2 researchers (A.H.L. and M.S.) using NVivo (NVivo Pro Enterprise). The codes were developed deductively (derived from quantitative domains) and inductively (derived from the interviews, discussions, and memos written after each interview) and created through an iterative process. The transcripts were coded concurrently by 2 researchers (A.H.L. and M.S.), with discussions and revisions to the codebook after each coded transcript until good agreement (κ = 0.73) was reached with the third transcript.^21^ The remaining 9 interviews were coded independently, with weekly discussions to address ambiguities until consensus was reached. Findings were validated through triangulation among us and a search for disconfirming evidence in subsequent interviews. In this way, using rigorous content and thematic analysis, we identified core categories and themes to produce trustworthy and insightful findings.^20^

#### Integrated Mixed-Methods Analysis

The surveys and interviews were integrated first by building the interview guide based on survey responses and second by using the survey data to direct the sampling selection for the interviews as depicted in the eFigure in the Supplement. Third, the interview data were merged with the survey data in joint displays to expand the understanding of the survey data with a visual aid that merged quantitative data, qualitative quotes and themes, and meta-inferences.^22^

## Results

### Cohort Characteristics

A total of 152 patients (mean [SD] age, 72.3 [4.4] years; 146 [96.0%] male) were surveyed and 12 patients (mean [SD] age, 72.3 [4.8] years; 11 [91.7%] male) were interviewed as part of the explanatory, sequential, mixed-methods study (Table 1). The median study follow-up period for each patient from the inpatient admission date to the survey date was 307 days (IQR, 265-344 days). The median total inpatient hospital length of stay during the follow-up period was 7 days (IQR, 4-16 days) for survey participants. Most patients had evidence of comorbidities in the Corporate Data Warehouse: 98 (64.5%) had visits for hypertension, 53 (34.9%) had visits for diabetes, and 23 (15.1%) had visits for chronic kidney disease. In the overall cohort, the mean (SD) home time was 94.3% (10.4%), and the median home time was 97.8% (IQR, 94.6%-98.6%). The mean (SD) time away from home was similar at skilled facilities or rehabilitation centers (2.4% [8.3%]) and at hospitals (3.3% [6.7%]). In the low OSS subcohort, the mean (SD) home time was 95.4% (10.4%), and the median home time was 98.6% (IQR, 96.3%-99.1%). In the high OSS subcohort, the mean (SD) home time was 93.2% (12.1%), and the median home time was 97.4% (IQR, 94.2%-98.8%). Both survey and interview samples were heterogenous and represented a variety of surgical specialties.

**Table 1.** Demographic and Comorbidity Information for Participants in the Quantitative Surveys and Qualitative Interviews^a^

Variable	Surveys (n = 152)	Interviews (n = 12)
Home time, %^b^		
Entire cohort		
Mean (SD)	94.3 (11.3)	85.2 (17.2)
Median (IQR)	98.2 (94.7-99.0)	92.9 (79.3-97.1)
LHT cohort (n = 6)^c^		
Mean (SD)	NA	72.9 (16.8)
Median IQR	NA	78.9 (67.6-81.4)
HHT cohort (n = 6)^c^		
Mean (SD)	NA	97.6 (1.4)
Median (IQR)	NA	97.4 (96.9-98.7)
Nonhome time, %		
SNF or rehabilitation time		
Mean (SD)	2.4 (8.3)	NA
Median (IQR)	0 (0-0)	NA
Hospitalization time		
Mean (SD)	3.3 (6.7)	NA
Median (IQR)	1.5 (0.9-3.1)	NA
Age, y		
Mean (SD)	72.3 (4.4)	72.3 (4.8)
Median (IQR)	72.0 (69.5-75.0)	72.0 (69.8-75.0)
Sex		
Female	6 (3.9)	1 (8.3)
Male	146 (96.1)	11 (91.7)
Mode of survey contact		
In-person	7 (4.6)	0
Telephone	145 (95.4)	12 (100)
Index surgery discharge location		
Home	119 (78.3)	9 (75.0)
SNF, rehabilitation center, or other facility	33 (21.7)	3 (25.0)
Operative stress score^d^		
Low	72 (47.4)	3 (25.0)
High	80 (52.6)	9 (75.0)
Specialty		
General surgery	27 (17.8)	2 (16.7)
Neurosurgery	14 (9.2)	2 (16.7)
Orthopedic surgery	66 (43.4)	4 (33.3)
Thoracic surgery	7 (4.6)	1 (8.3)
Urology surgery	14 (9.2)	1 (8.3)
Vascular surgery	24 (15.8)	2 (16.7)
Race		
American Indian or Alaska Native	2 (1.3)	0
Asian	4 (2.6)	1 (8.3)
Black or African American	11 (7.2)	1 (8.3)
Native Hawaiian or Pacific Islander	1 (0.7)	0
White	117 (77.0)	9 (75.0)
Unknown	17 (11.2)	1 (8.3)
Ethnicity		
Hispanic or Latino	22 (14.5)	1 (8.3)
Not Hispanic or Latino	124 (81.6)	11 (91.7)
Unknown	6 (4.0)	0
Comorbidities		
Diabetes	53 (34.9)	NA
Hypertension	98 (64.5)	NA
Hyperlipidemia	18 (11.8)	NA
Congestive heart failure	20 (13.2)	NA
Chronic kidney disease	23 (15.1)	NA
COPD	21 (13.8)	NA

Abbreviations: COPD, chronic obstructive pulmonary disease; HHT, high home time; LHT, low home time; NA, not applicable; SNF, skilled nursing facility.

^a^ Data are presented as number (percentage) of participants unless otherwise indicated.

^b^ Home time was measured as the percentage of days at home from the surgery date to the survey date.

^c^ Interview participants only.

^d^ Low operative stress scores were 1 and 2, and high were 3 to 5.

### Quantitative Survey Findings

The association between home time and patient-reported outcomes (QoL, functional status, and decisional regret) are presented in Table 2. A higher proportion of home time was associated with higher physical QoL (VR-12 PCS: *r* = 0.33; 95% CI, 0.18-0.47; *P* < .001) and functional status (ADL score: *r* = 0.21 95% CI, 0.06-0.36; *P* = .008; IADL score: *r* = 0.21; 95% CI, 0.04-0.37; *P* = .009). The CASP-19 control subscale was weakly correlated with home time (*r* = 0.13; 95% CI, −0.03 to 0.29; *P* = .11). The other CASP-19 domains, the MCS of the VR-12, and the DRS were not associated with home time in quantitative analysis.

**Table 2.** Correlation of Home Time With Function, Quality of Life, and Decisional Regret in the 152 Survey Participants

Variable	Mean (SD)	Median (IQR)	Overall cohort		Low OSS subcohort^a^		High OSS subcohort^a^	
			*r* (95% CI)^b^	*P* value	*r* (95% CI)^b^	*P* value	*r* (95% CI)^b^	*P* value
**Quality of life**								
VR-12								
Physical component score	32.8 (11.5)	32.9 (22.8-41.7)	0.33 (0.18 to 0.47)	<.001	0.39 (0.12 to 0.53)	.001	0.33 (0.16 to 0.47)	.003
Mental component score	50.8 (11.6)	53.0 (45.1-59.6)	0 (−0.17 to 0.16)	.97	0.13 (−0.09 to 0.3)	.28	−0.12 (−0.35 to 0.12)	.27
CASP-19 score								
Total	40.3 (8.8)	41.0 (35.0-47.0)	0.11 (−0.05 to 0.26)	.18	0.2 (−0.02 to 0.37)	.09	0.02 (−0.19 to 0.25)	.87
Control	7.2 (2.7)	7.0 (5.0-9.0)	0.13 (−0.03 to 0.29)	.11	0.16 (−0.12 to 0.36)	.17	0.10 (−0.08 to 0.26)	.39
Autonomy	9.5 (2.9)	10.0 (8.0-12.0)	0.06 (−0.09 to 0.20)	.48	0.13 (−0.09 to 0.3)	.26	−0.02 (−0.23 to 0.18)	.86
Self-realization	13.1 (2.6)	14.0 (12.0-15.0)	0.03 (−0.15 to 0.17)	.76	0.18 (−0.04 to 0.41)	.14	−0.12 (−0.37 to 0.12)	.28
Pleasure	10.5 (3.1)	11.0 (9.0-13.0)	0.09 (−0.07 to 0.25)	.26	0.19 (−0.03 to 0.39)	.12	0.01 (−0.21 to 0.21)	.96
**Functional status surveys**								
Activities of daily living	5.8 (0.5)	6.0 (6.0-6.0)	0.21 (0.06 to 0.36)	.008	0.26 (0.04 to 0.42)	.03	0.15 (−0.04 to 0.39)	.19
Instrumental activities of daily living	7.6 (0.9)	8.0 (8.0-8.0)	0.21 (0.04 to 0.37)	.009	0.12 (−0.14 to 0.33)	.31	0.25 (0.03 to 0.47)	.02
**Decisional regret survey**								
Decision Regret Scale score	7.6 (15.1)	0.0 (0.0-10.0)	−0.04 (−0.21 to 0.13)	.64	0.11 (−0.09 to 0.34)	.36	−0.22 (−0.47 to 0.04)	.047

Abbreviations: CASP-19, 19-Item Control, Autonomy, Self-realization, and Pleasure scale; OSS, operative stress score; VR-12, Veterans RAND 12-item Health Survey.

^a^ Low OSSs were 1 and 2, and high were 3 to 5.

^b^ Spearman rank correlation.

In the stratified analysis, physical QoL was similarly associated with home time in the low OSS (*r* = 0.39; 95% CI, 0.12-0.53; *P* = .001) and high OSS (*r* = 0.33; 95% CI, 0.16-0.47; *P* = .003) subcohorts. Activities of daily living and IADLs were associated with home time in both the low and the high OSS subcohorts, respectively. In the low OSS subcohort, ADL score was associated with home time (*r* = 0.26; 95% CI, 0.04-0.42; *P* = .03), whereas in the high OSS subcohort, IADL score was associated with home time (*r* = 0.25; 95% CI, 0.03-0.47; *P* = .02). Decisional regret was inversely associated with home time in the high OSS subcohort only (*r* = −0.22; 95% CI, −0.47 to −0.04; *P* = .047).

### Qualitative and Integrated Findings

Qualitative themes are shown with quantitative findings as joint displays in the Figure and in Table 3 and Table 4.^22^ First, despite reporting receipt of excellent service at rehabilitation facilities, patients in both the HHT and LHT groups described a strong desire to be discharged home as soon as possible (eg, “It was all right [at rehabilitation facility]. It just wasn’t home”). Patients uniformly viewed the transition to home as a milestone in the recovery process. They perceived being home as a physical and psychological indicator of recovery. Patients indicated that home was the default place for recovery regardless of type of surgery or percentage of recovery time spent at home. Second, although patients preferred to recover at home, some acknowledged that staying at a rehabilitation facility could be beneficial, in particular when specialized medical assistance was needed. Third, many patients identified home with independence. If help was needed, patients with HHT were more likely to disclose needing help with yardwork and higher-level tasks rather than with ADLs, such as toileting. Fourth, patients who lived alone described valuing time recovering at a rehabilitation facility more than did those who lived with or near an informal caregiver.

**Figure.**
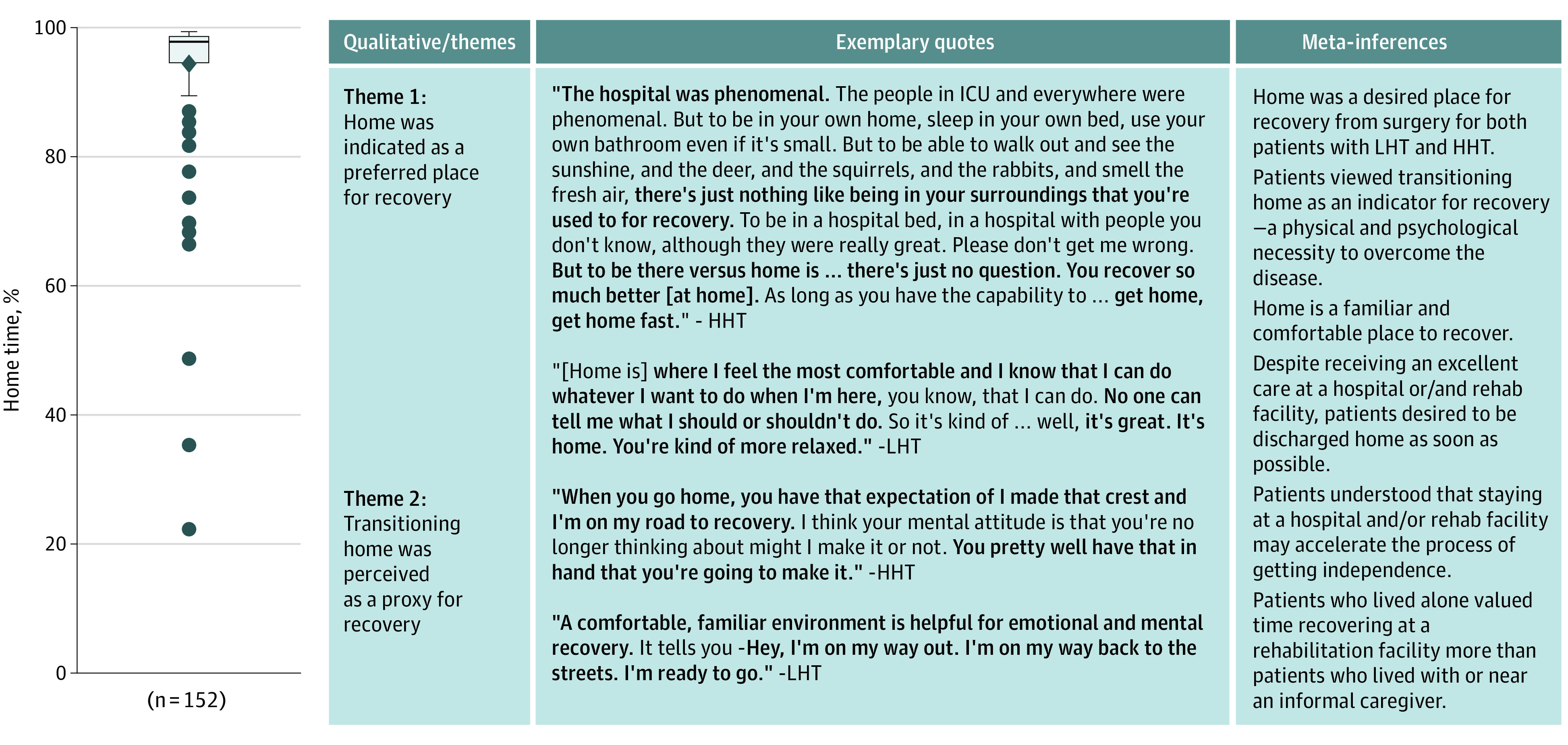
Joint Display of Home Time Distribution and Qualitative Themes Regarding Importance of Home Time in Postoperative Recovery Patients had a mean (SD) home time of 94.3% (10.4%), with a range of 22.2% to 99.5% and a median of 97.8% (IQR, 94.6%-98.6%). HHT indicates high home time; ICU, intensive care unit; and LHT, low home time.

**Table 3.** Joint Display of Quantitative Association of Quality of Life With Home Time, Representative Qualitative Interview Quotations, and Meta-inferences for 151 Individuals

Variable	Quantitative results	Qualitative interview quotes (subgroup)	Meta-inferences
VR-12			
PCS	Positive correlation with home time	“Oh, before the surgery I was in a lot of pain…it was very hurtful for me to walk at all....And now, I’m walking fine.” (HHT) “Just a little pain and, you know, it hasn’t gotten well all the way yet…I think it went down to my leg….I can walk better now, but I don’t know.” (LHT)	Pain levels are an indicator for the PCS score. These 2 examples demonstrate the larger trend that physical health improved 6 to 12 mo after surgery among patients with HHT and more patients with LHT were still experiencing more pain and lingering impairment.
MCS	No correlation with home time	“It can become depressing. But you’ve got to be cognizant of that and you need to fight it and say, ‘Okay, every day is going to get better.’” (HHT) “My concentration is actually good…my biggest hobby is playing Bridge….I play at a high level of Bridge. I may not be playing quite as well as I was before all this happened, but I’m still playing at a pretty high level.” (LHT)	The MCS (eg, mental health) had no association with home time, and the interviews provided similar examples from patients with HLT and LHT. The VR-12 asks how patients feel today 6 to 12 mo after surgery. The connection may have been stronger if the survey was administered closer to hospital discharge.
CASP-19	No overall correlation with home time	“The doctors asked me to go to rehab[ilitation],…but I was so tired of being in there [hospital]. They put me on 3 days bed rest, and I thought that was just laying in bed.…So I said, ‘No, I want to go home. That’s going to be my rehab[ilitation].’” (HHT) “Well, if it had been up to me, I would probably have said, ‘No, I’ll go straight home.’ But they wanted me to have some more supervision….If that’s what they want, I’ll go.…I was laying in bed here or laying in bed at home…So I could have done a lot of it at home. I was definitely ready to leave when my 2 weeks were up.” (LHT)	Occasionally, patients asserted a preference for a desired location; most often the preference was to go home (vs to a rehabilitation facility). Both examples here show patients with a desire to go home after their surgery despite their physicians recommending they go to a rehabilitation facility. There may be a weak correlation between control and home time owing to patients with HHT being more likely to assert their preferences and patients with LHT being more likely to defer to physician preference, relinquishing their control.

Abbreviations: CASP-19, 19-Item Control, Autonomy, Self-realization, and Pleasure 19-item scale; HHT, high home time; LHT, low home time; MCS, mental component score; PCS, physical component score; VR-12, Veterans RAND 12-item Health Survey.

**Table 4.** Joint Display of Quantitative Association of Functional Status and Decisional Regret With Home Time, Representative Qualitative Interview Quotations, and Meta-inferences

Variable	Quantitative results	Qualitative interview quotations (subgroup)	Meta-inferences
Functional status (n = 152)			
Activities of daily living	Positive correlation with home time	“I felt great [at home]….I was real sore getting in and out of bed, about less than a month. After that, I could get up and in and out of bed, no problem. Go in the kitchen, fix my own breakfast, and everything. But I just couldn’t go out and mow the lawn,…so I had a young man down the street do all the yardwork for me.” (HHT)	The patients who spent more time at home were generally more independent. The types of activities requiring assistance for patients with HHT tended to be complex tasks, whereas patients with LHT were more likely to need assistance with routine activities after transitioning home.
Instrumental activities of daily living	Positive correlation with home time	“There was an electrical problem with my washer and dryer. I had to go to a laundromat to do my laundry.…I had to do more laundry because I was having trouble with leaking colostomy bags….That was a problem with my whole recovery. The worst part of my recovery was my colostomy bag” (LHT).	
Decision Regret Scale score (n = 151)	No correlation with home time	“[The surgeon] leaned towards the invasive surgery versus the stents….It was a decision fully between the three of us, the doctor and my wife and I.” (HHT) “If I didn’t get the infection that caused all of the problems, because my expectation of the original surgery was to be a success, and of course it wasn’t a success, that was kind of hard to go through. But I would do it all over again…because my incontinence was so bad, I mean, I would definitely do it no matter what you said. This was the last hope, you know, of becoming somewhat normal.” (LHT)	Most patients did not regret the surgery regardless of whether they felt like they were part of the decision to have surgery or whether the surgery was considered “successful.” The patients often considered the alternative to be chronic pain or disability and/or death and therefore did not regret choosing the surgical intervention.

Abbreviations: HHT, high home time; LHT, low home time.

In confirmation of the quantitative survey findings, interview respondents with more time at home were more likely to report and describe physical improvement in postoperative recovery compared with respondents who spent less time at home (Table 3). Conversely, although mental health–related QoL (MCS) was not associated with home time in surveys, interview respondents frequently discussed how being at home and in familiar surroundings facilitated mental and social recovery. With respect to locus of control, interview respondents with LHT generally deferred to practitioner recommendations more often than patients with HHT in choosing a discharge location.

The HHT interview group generally described higher independent functional status in ADLs and IADLs (Table 4). Overall, there was low decisional regret in the entire cohort, and decisional regret about choosing surgery was not associated with home time in general. Interview respondents explained that they considered the alternative to surgery to be chronic pain or death. Therefore, even after unexpected complications, patients did not regret the decision to undergo an operation. The significant inverse association of home time with decisional regret in patients with high OSSs could not be analyzed in qualitative interviews because of the small sample size.

## Discussion

This novel mixed-methods study demonstrated a statistically significant correlation of home time with physical QoL and independent functional status among US veterans after inpatient surgery. In qualitative interviews, a primary theme was emotional or social recovery associated with time spent at home, suggesting the value of return to home as a surrogate for recovery for patients. Our findings suggest that home time is a promising, patient-oriented outcome measure for surgical recovery that warrants further study as a quality measure.

Although there is minimal extant literature in surgery assessing time spent at home in the first year after major surgery, in an analysis of Medicare patients undergoing cystectomy, pancreaticoduodenectomy, gastrectomy, and esophagectomy, Suskind et al^10^ demonstrated that patients who spent more time at home in the first year after cancer surgery were more likely to survive to 1 year. Specifically, patients alive at 1 year spent 13% of that year away from home, and patients who had died by 1 year spent 25% of that year away from home. No deaths occurred in the sample in the present study because we included only patients alive 6 months after surgery, and at 1 year, patients spent a mean of 94.3% of their time at home. This difference may have been the result of the sample of patients being from various specialties (general surgery, vascular surgery, orthopedic surgery, urology surgery, thoracic surgery, and neurosurgery) and not just from surgical oncology. The association of home time with stroke severity and functional status measures has been shown in patients with stroke.^8^ In that study, higher Glasgow Coma Scale scores, indicating higher levels of consciousness, were moderately and significantly correlated with more home time among patients with ischemic stroke (*r* = 0.35) and hemorrhagic stroke (*r* = 0.39). Functional independence scores were highly correlated with home time (*r* = 0.63-0.69), and similar to our results, physical and motor scores were more strongly correlated with home time than cognitive scores (r = 0.62-0.68 vs 0.36-0.47).

Our study did not show an association of home time with mental or emotional QoL in quantitative data. However, there was a prevalent theme throughout the qualitative interviews indicating that home was a safe space for mental and emotional well-being and for social support. Home was the preferred place for recovery universally regardless of time spent at home or patients going to a rehabilitation center by choice. The patients viewed returning home as a marker or proxy for recovery. The lack of association in our study may be attributable to the VR-12 MCS for QoL, which measures how patients are doing the day they are interviewed rather than during the whole recovery process. No mental or emotional QoL survey measures are available in surgery that focus on the type of emotions reported in the interviews. Our findings highlight the lack of suitability for existing QoL scales for assessing mental and emotional postoperative recovery, and better QoL measures may be needed to assess this outcome in patients undergoing surgery.

The study participants overall reported low decisional regret scores, and in the entire cohort, we did not find an association with home time. However, there was an inverse association of decisional regret with home time in the high OSS subcohort. The high OSS subcohort also had lower home time, suggesting that after more complex procedures that may have longer expected recovery time, time away from home may contribute to decisional regret regarding the decision for initial surgery. The qualitative cohort was purposively sampled for low and high home time; thus, we could not analyze this finding in the qualitative data. This potential association is worth exploring in future research because it may be an opportunity to improve the informed consent and discharge planning and communication process for setting realistic expectations about postoperative recovery and to minimize regret.

Home time can be easily collected using administrative data sets, such as the Corporate Data Warehouse or Medicare, and has several advantages in surgical health services research. Functional and QoL measures are difficult to collect in registry and administrative data sets in large surgical cohorts. Their validity is also threatened by interrater variability, loss to follow-up of research participants, or limitations in collecting primary data from patients vs caregivers. Home time may serve not only as a proxy or surrogate for function and QoL measures for large database research but also as a covariate for measuring quality of care and health care value at the patient and hospital levels. For instance, in a recent study^5^ of patients with heart failure, the authors calculated hospital-level, risk-adjusted, 30-day home time (in days) using administrative claims data from 100% Medicare hospitalizations and showed that risk-adjusted 30-day home time was significantly associated with 30-day readmission and mortality rates. Of more importance, the authors were able to meaningfully reclassify one-third of the hospitals in their performance status. Hospitals that had high 30-day risk-adjusted readmission rates and low 30-day home time days had higher inpatient and 30-day mortality and vice versa. This finding is particularly relevant for surgical performance and quality measures because higher readmission rates do not account for the competing risk of mortality; therefore, hospitals that have high surgery-related readmission rates but a low surgical mortality can theoretically be penalized. The time horizon for home time (approximately 6 months to 1 year after surgery) may also be advantageous as a hospital performance metric for surgical outcomes because it would account for hospitals optimizing postacute care and ensuring recovery to presurgery levels of physical function and safe return to home without subsequent readmissions. However, caution would be needed during development of home time as a quality metric to ensure that patients without caregiver support or those who would benefit from intensive rehabilitation for physical recovery are not adversely impacted.

### Limitations

This study has limitations. First, as in all survey and qualitative studies, representative sampling and sample size were limited. Our findings therefore should be interpreted as pilot data intended to develop larger studies to test generalizability and to inform the utility of home time as a quality measure of patient-centered surgical care. Second, home time in the study was not adjusted for any covariates because of the small sample size, and we anticipate conducting future studies to understand variability in home time across specialties and risk-adjustment for important covariates. Third, the lack of association of home time with mental QoL and other domains of control and autonomy may be attributable to low statistical power or the limitations of the instruments used (VR-12 and CASP-19) because our qualitative data provided thematic support of an association of home time with mental and emotional recovery, which should be further tested quantitatively. Fourth, our study’s generalizability is also somewhat limited because participants were nearly all male given their predominance in the veteran population. Further study is needed to confirm the association of home time with function and QoL among women. Fifth, this was a retrospective sampling of patients who were alive between 6 months and 1 year after surgery, skewing our home time data to the right. To address the variable time span from surgery to survey date, we used percentage home time as a means of standardization.^23^ However, we were missing important QoL and functional status information for people who may have had lower home time and died within 6 months of their operation. In future prospective studies, we will be able to measure QoL and function in patients who died within 6 months and use actual days spent at home at the 180-day time point after surgery.

## Conclusions

In this mixed-methods study including a survey and qualitative interviews, we found that HHT in the first year after inpatient surgery was associated with improved function and physical QoL among US veterans. Patients indicated that they considered home as the preferred place for postoperative recovery, and qualitative interviews suggested the importance of home time in mental and emotional recovery from surgery. Our data provide important foundational insights into the use of home time, a primarily administrative data measure, as a patient-centered quality metric for surgical research.

## Notes

Supplement. **eFigure.** Home Time Mixed-Methods Explanatory Sequential Study Design **eTable.** List of ICD-9 and ICD-10 Codes for Comorbidity Classification
