# Supplementary material for: Perspectives on Home Time and Its Association With Quality of Life After Inpatient Surgery Among US Veterans
Source: JAMA Netw Open. 2022 Jan 11;5(1):e2140196. doi: 10.1001/jamanetworkopen.2021.40196 (PMC8753502; doi:10.1001/jamanetworkopen.2021.40196)
Supplement: Supplement. — eFigure. Home Time Mixed-Methods Explanatory Sequential Study Design eTable. List of ICD-9 and ICD-10 Codes for Comorbidity Classification [file jamanetwopen-e2140196-s001.pdf]

Supplementary Online Content

Arya S, Langston AH, Chen R, et al. Perspectives on home time and its association with quality of life after inpatient surgery among US veterans. *JAMA Netw Open*. 2022;5(1):e2140196. doi:10.1001/jamanetworkopen.2021.40196

eFigure. Home Time Mixed-Methods Explanatory Sequential Study Design

eTable 1. List of ICD-9 and ICD-10 Codes for Comorbidity Classification

This supplementary material has been provided by the authors to give readers additional information about their work.

© 2022 Arya S et al. *JAMA Network Open.*

eFigure. Home Time Mixed-Methods Explanatory Sequential Study Design

QUAN → Explained by → QUAL → DATA INTEGRATION

QUAN

Quantitative Data

Chart Review

Home-time

Home-time Percentage

Surveys

Quality of Life

VR-12 PCS

VR-12 MCS

CASP-19

- Control*
- Autonomy
- Self-reliance
- Pleasure

Functional Status

ADLs

IADLs

Surgery Decisional Regret

DRS

Build Interview Guide

Connect for Sampling

QUAL

Qualitative Interview Guide Design

The word “home” can mean very different things to different people. What does “home” mean to you?

How did recovering at home compare to recovering at __{rehab}____?

There are multiple aspects of recovery like physical, emotional, cognitive, social… How did being at (home/rehab) affect your physical recovery from surgery?

How did being at (home/rehab) affect other than physical / or mental/emotional aspects of your recovery from surgery?

How much of a say did you have in where you went when you left the hospital? (Home vs Non-home)

How much control did you have over {your recovery process}?

What are the major differences in your life now compared to before the surgery?

What, if anything, would you change about your surgery and recovery process?

DATA INTEGRATION

Merging Domains and Interviews

Codes

- Control
- Discharge
- Expectation
- Home-meaning
- Information
- Self-assessment

Themes

Home was indicated as a preferred place for recovery.

Transitioning home was perceived as a proxy for recovery.

Joint Displays

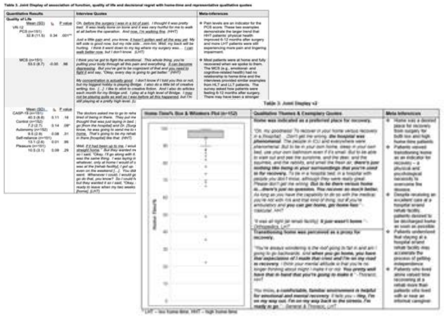

© 2022 Arya S et al. *JAMA Network Open.*

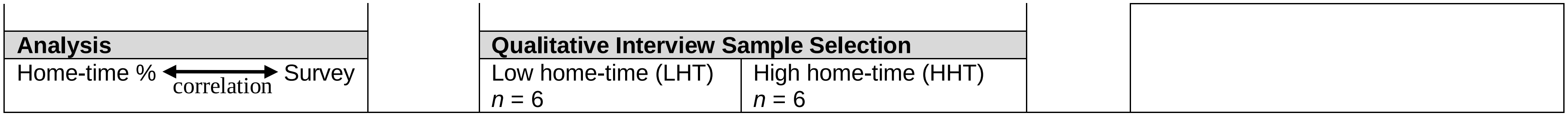

Note*=The “Control” subscale the highest association with home-time Percentage which indicated a potential trend, so questions were added to the interview guide to explore this domain. The other CASP-19 subscales were not specifically addressed

eTable. List of ICD-9 and ICD-10 Codes for Comorbidity Classification

© 2022 Arya S et al. *JAMA Network Open.*

Comorbidities	ICD-9	ICD-10
Diabetes	249.x, 250.x	E08.x, E09.x, E10.x, E11.x, E13.x
Hypertension	401.0, 401.1, 401.9	I10.x
Hyperlipidemia	272.2, 272.4	E78.2, E78.4
Congestive heart failure	398.91, 428.0 - 428.9	I50.1, I50.2, I50.3, I50.4, I50.9, I50.8, I09.81
Chronic kidney disease	585.1, 585.2, 585.3, 585.4, 585.5, 585.6, 586	V420, V451, V560 - V5632, V568, V4511 - V4512, N18.1, N18.2, N18.3, N18.4, N18.5, N18.6, N19, Z4901, Z4902, Z4931, Z4932, Z9115, Z940, Z992
Chronic obstructive pulmonary disease	490, 491.0, 491.1, 491.2, 491.8, 491.9, 492.0, 492.8, 493, 494.0, 494.1, 495, 496	J40, J41.0, J41.1, J41.8, J42, J43.0, J43.1, J43.2, J43.8, J43.9, J44.0, J44.1, J44.9, J45, J47, J67

© 2022 Arya S et al. *JAMA Network Open.*
